# Psychological problems and quality of life of patients with oral mucosal diseases: a preliminary study in Chinese population

**DOI:** 10.1186/s12903-018-0696-y

**Published:** 2018-12-27

**Authors:** Chao Yang, Lina Liu, Huijie Shi, Yuanyuan Zhang

**Affiliations:** Department of General Dentistry, The First Affiliated Hospital of Zhengzhou University, No. 1 Jianshe East Road, Zhengzhou City, China

**Keywords:** Oral health-related quality of life, Psychological problems, Recurrent aphthous ulcers, Oral lichen planus, Burning mouth syndrome

## Abstract

**Background:**

Psychological problems might play important roles in oral mucosal diseases such as recurrent aphthous ulcers (RAU), oral lichen planus (OLP), burning mouth syndrome (BMS), but the relevance to patients’ quality of life remained controversial. The aim of this study was to investigate the psychological problems and oral health-related quality of life in patients with RAU, OLP, and BMS in China, to assess the relationship between psychological problems and quality of life.

**Method:**

Thirty-nine RAU patients, 45 OLP patients, 15 BMS patients and 45 healthy controls were enrolled in the study. Hospital Anxiety and Depression Scale (HADS) were chosen to analyze the patients’ psychological problems. Oral Health Impact Profile (OHIP-14) was used to measure the OHRQoL. The scores of HADS and OHIP-14 were used to analyze the relationship between psychological problems and the quality of life of oral mucosa patients.

**Results:**

Each of OHIP-14 scores and HADS scores in RAU, OLP, BMS was higher than the control group, and there was significant difference in the patients groups with the control cases(*P* < 0.05). OHIP-14 score of RAU was the highest in three patient groups. Its OHRQoL was lowest in the three groups, which had statistical significance (*P* < 0.05). Positive correlations existed between the psychological problems and the quality of life of the three patient groups (*r*_*s*_ > 0, *P* < 0.05), except for the depression of the BMS group (*r*_*s*_ = 0.168, *P* = 0.395).

**Conclusion:**

Patients with oral mucosal diseases such as RAU, OLP, and BMS had higher levels of anxiety, depression, and lower quality of life. The patient’s psychological problems were related to their quality of life, suggesting that the psychological state of patients with oral mucosal disease need more attention.

## Background

Oral mucosal diseases were series of chronic diseases in the oral cavity, and they often manifested different lesions on the oral mucosa under the influence of various factors. Recurrent aphthous ulcers (RAU) [1], oral lichen planus (OLP) [2], burning mouth syndrome (BMS) [3] appeared more common,chronic, and recalcitrant. As we all know, the incidence of RAU, OLP and BMS surveys varied greatly among different regions and populations. The incidence of global RAU was as high as 4.0% [1]. RAU was more common in adults, more women. What’s more, the high-risk groups were the people who was under 40, Caucasian, non-smokers and with better socioeconomic status [4]. The incidence of OLP was between 0.9 and 2.2% [2]. OLP was more common in adults, especially in women, 50–55 years old [5]. In addition the incidence of BMS was between 0.7 and 4.6%, which occurs in menopausal women aged 50–70 [3]. The clinical manifestion such as pain can affect the daily life of patients, and had a great negative impact on the quality of life of patients [6]. At present, the treatment of oral mucosal diseases mainly focused on lesion treatment and relieves symptoms [7]. Due to their many influencing factors, the therapeutic effect on oral mucosal diseases was limited.

Oral mucosal diseases as RAU, OLP, and BMS had many causes, which researchers had not been able to fully explain [1–3]. Studies had shown that the occurrence of RAU, OLP, BMS might be related to psychological factors. Because the oral mucosa was very responsive to emotional effects such as stress, anxiety and depression; oral disease might be a direct manifestation of emotions, or it might be an indirect result of psychological changes [8], but this conclusion had not been clearly confirmed so far. Many studies had evaluated different aspects of oral mucosal disease and found that RAU recurred under stress. Psychological problems and their relationship with skin diseases had been fully recognized [9]. Psychological factors appeared more important in oral mucosal diseases than cutaneous diseases. However, the importance in oral lichen planus (OLP) remained controversial. Research reported [10] more than 50% of BMS can be explained by psychological factors, but the causal and psychological factors of BMS so far were still unclear. In addition, most current studies had shown that anxiety was associated with these diseases, but depression was controversial.

Although psychological problems had a wide impact on oral mucosal diseases, psychological problems had not been taken seriously in the treatment of oral mucosal diseases because of the lack of definitive evidence and regional differences in the treatment of oral mucosal diseases. The treatment of oral mucosal diseases was in the symptomatic treatment stage. It did not help the improvement of patients’ quality of life. The purpose of this study was to investigate the psychological problems and oral health-related quality of life (OHRQoL) in patients with RAU, OLP, and BMS, to assess the relationship between psychological problems and quality of life, to seek new ways to improve the efficacy of oral mucosal diseases, and to improve the quality of life of patients with oral mucosa.

## Methods

Ninety-nine patients (from January 2017 to August 2017) with RAU, OLP and BMS were enrolled in the study at the Department of Oral Medicine, Stomatology Hospital of Henan province, including 39 RAU, 45 OLP and 15 BMS. The study was approved by the local ethics committee, the Medical Research Ethics Committee of the First Affiliated Hospital of Zhengzhou University. The interviewer (before trained) gave the patients detailed information about the study, and the interview was carried out after receiving informed consent from the patients.

Inclusion criteria to take part in the study were: The first-time patients who complained of oral diseases had no history of mental disease, such as the diseases diagnosed with epilepsy, depression, anxiety, symptoms of neurological disorders caused by other diseases or disturbance of consciousness in their previous medical history. The patients were also required to be accompanied by no cutaneous manifestations of the diseases studied. They had good cognitive abilities, aged 18 years, diagnosed with RAU, OLP, or BMS, according to the clinical symptoms, medical history and blood examination and other auxiliary examination [8, 11]. What’s more, patients had a detailed knowledge of the disease and agreed to participate in the investigation. The knowledge of the disease included the concept of the disease, the hazards, the influencing factors in daily life, and the treatment options. Exclusion criteria were: not wishing to take part in the study, took anxiolytic and phylogenetic during the previous six months, suffered from systemic disease. Forty-five healthy controls came from the subjects who resorted to our hospital for health examinations without local or systemic disease or took anxiolytic and thermostatic. In addition, the experiment required that the age-sex ratio of the health control group to the experimental group be matched as much as possible.

The interview consists of Oral Health Impact Profile (OHIP-14) [12], Hospital Anxiety and Depression Scale (HADS) [13, 14]. The interview was taken when the patient was first diagnosed, and the condition was stable.

The OHIP-14 consists of 14 items, each item is scored: “never”-score 0. “hardly ever”-score 1, “occasionally”-score 2, “often”-score 3, “very often”-score 4. The OHIP-14 is divided into 7 different domains: “functional limitation” (item1,2); “physical pain” (item3,4); “psychological discomfort” (item5,6); “physical disability” (item7,8); “psychological disability” (item9,10); “social disability” (item11,12); “handicap” (item13,14), Omit OHIP score is 56. In this model, the higher scores show a poorer state of oral health.

The HADS is 14 –item measures consist of 7 anxiety items (HADS-A) and 7 depression items (HADS-B). All items are coded on a four-point scale from 0 (not at all) to 3 (most of the time). On the basis of their scores, individuals can be categorized into three score ranges: normal (0–7), borderline abnormal (8–10) and abnormal (11–21), score over 8 is positive.

### Statistical analysis

All analyses were conducted in SPSS 21.0. We used Kruskal-Wallis test for quantitative data and rank sum test for qualitative data (more than two samples). Spearman’s correlation coefficients were examined to determine the correlations between OHIP-14 and HADS. Probability of *P* < 0.05 was accepted as significant.

## Results

Table 1 shows: there are 39 cases of RAU group, 25 males and 14 females, aged 20–63 years, average age of 37.05, and male female: 1.79:1.OLP group had a total of 45 people, male 17, female 28, aged 20 to 73 years old, average age of 47.22, gender ratio male: 0.61 1:1.The BMS group had 15 people, 8 men, 7 women, between 32 and 80 years old, with an average age of 60.53, and the sex ratio was male:female: 1.14:1.There were 45 cases in the control group, 14 cases of males and 35 females, aged 23–70 years, with an average age of 37.05 and a female ratio of 0.45:1. Regarding the HADS-A, we found significant differences among the groups, the OLP, RAU, BMS group had significant difference with healthy controls, healthy controls and OLP group (*Z* = 4.441, *P* < 0.001), healthy controls and RAU (*Z* = 4.444, *P* < 0.001), healthy controls and BMS group (*Z* = 3.091, *P* = 3.091). And HADS-D, we found significant differences between the groups: healthy controls and OLP group (*Z* = 3.689, *P* < 0.001) and healthy controls and RAU group (*Z* = 3.945, *P* = 3.945) and healthy controls and BMS group (*Z* = 3.451, *P* = 3.451).

**Table 1:** Participants’ demographic statistics and their anxiety and depression status statistics in the Study

	Age	Sex	Anxiety						Depression				
		Male (case)	Female (case)	Normal (case)	Borderline abnormal (case)	Abnormal (case)	*Z*	*P*	Normal (case)	Borderline abnormal (case)	Abnormal (case)	*Z*	*P*
Healthy control group	36.04 ± 12.98	14	31	43	1	1			43	2	0		
OLP	47.22 ± 12.70	17	28	26	8	11	−4.441	0.000*	28	10	7	−3.689	0.000*
RAU	37.05 ± 12.03	25	14	25	8	6	−4.444	0.000*	25	8	6	−3.945	0.000*
BMS	60.53 ± 11.88	8	7	9	2	4	−3.091	0.002*	10	1	4	−3.451	0.001*

Note: *P < 0.05

For the OHIP-14 score, Table 2 shows the significant differences between the groups. In all item, the OLP group (P50 = 13,000, not shown in the table) was higher than the healthy control group (P50 = 9.000), *Z* = − 2.113, *P* = 0.033.There was a significant difference in social disability (*Z* = − 2.965. *P* = 0.003). All items in the RAU group (P50 = 22.000) was above the healthy control group (P50 = 9.000), *Z* = − 4.327, *P* < 0.001. Significant differences were found between groups in each domain. All item of BMS group (P50 = 12.000) was higher than the healthy control group (P50 = 9.000). *Z* = − 0.915, *P* = 0.360. There was a significant difference between the two groups: physical disability (*Z* = − 2.169, *P* = 0.030) and disability (*Z* = − 2.128, *P* = 0.033).

**Table 2:** Oral health impact profile (OHIP-49) sub-scale and all items, by healthy control group, OLP group, RAU group, and BMS group

	Healthy control *N* = 45	OLP N = 45			RAU *N* = 39			BMS *N* = 15		
	P_50_	P_50_	*Z*	*P*	P_50_	*Z*	*P*	P_50_	*Z*	*P*
All items	9.000	13.000	−2.133	0.033*	22.000	−4.327	0.000*	12.000	−0.915	0.360
Functional limitation	0.000	1.000	−1.332	0.183	3.000	−4.347	0.000*	0.000	−0.670	0.503
Physical pain	3.000	4.000	−1.849	0.064	4.000	−3.084	0.002*	3.000	−0.484	0.628
Psychological discomfort	2.000	2.000	−1.840	0.066	4.000	−3.260	0.001*	2.000	−0.893	0.372
Physical disability	2.000	2.000	−0.450	0.653	3.000	−2.640	0.008*	0.000	−2.169	0.030*
Psychological disability	1.000	1.000	−0.457	0.648	3.000	−4.035	0.000*	2.000	−1.511	0.131
Social disability	0.000	1.000	−2.965	0.003	2.000	−4.512	0.000*	1.000	−1.471	0.141
Handicap	0.000	1.000	−1.775	0.076	2.000	−2.616	0.009*	2.000	−2.128	0.033*

Note: *P < 0.05

Table 3 shows the correlation analysis results of OHIP-14 and HADS. In the healthy control group, oral health-related quality of life was positively correlated with anxiety (*r*_*s*_ = 0.289, *P* = 0.009) and depression (*r*_*s*_ = 0.277, *P* = 0.013). In RAU group, oral health related quality of life was positively correlated with anxiety (*r*_*s*_ = 0.463, *P* = 0.009) and depression (*r*_*s*_ = 0.408, *P* = 0.000). In OLP group, the life quality associated with oral health was positively correlated with anxiety (*r*_*s*_ = 0.449 *P* = 0.000) and depression (*r*_*s*_ = 0.500, *P* = 0.000). The oral health related life quality of BMS group was positively correlated with anxiety (*r*_*s*_ = 0.419, *P* = 0.000), but there was no correlation with depression (*r*_*s*_ = 0.168, *P* = 0.395).

**Table 3:** HADS score and OHIP-14 score correlation analysis in healthy control group, OLP group, RAU group and BMS group

	Healthy control group		RAU		OLP		BMS	
	*r*_*s*_	*P*	*r*_*s*_	*P*	*r*_*s*_	*P*	*r*_*s*_	*P*
OHRQoL and Anxiety	0.289	0.009*	0.463	0.009*	0.449	0.000*	0.419	0.000*
OHRQoL and Depression	0.277	0.013*	0.408	0.000*	0.500	0.000*	0.168	0.395

Note: *P < 0.05

## Discussion

With the rapid development of China’s economy in recent years, people’s quality of life and oral health awareness were improving gradually. As people’s life rhythm speeding up, the pressure of chinese people was growing. The psychological state was becoming more terrible. Anxiety and depression were two common negative emotions. Anxiety referred to the irritability and other emotions generated by the patient, while depression referred to the negative and low emotions generated by the patient, and anxiety was usually manifested earlier than depression [15]. Both of them will have a negative impact on the normal life of the person and even affect the physical health of the patient [8]. On this basis, people’s incomplete cognition and the fear of oral mucosal disease aggravated the bad psychological state. Thus the condition of psychological state might affect the oral mucosa disease. Based on the existing literature, oral health problems could result in pain and discomfort and could lead to problems in eating, interpersonal relationships, appearance and an individual’s positive self-image.

Because of the short experimental time, lack of specific patient observation time, case samples collected less, this study was only a preliminary investigation, pending further epidemiological investigation of larger samples and longer observation time. Nevertheless, the results obtained in this study had obvious significance.

Studies had shown that RAU happens to adults, more famale, under the age of 40, non-smokers, good social and economic status for high-risk groups [4]. The RAU group of patients in this study with an average age of 36, consistent with previous research, but the sex ratio of 1.79:1, contrary to the disease occurred in female, this has to do with this experiment materials in China, which has more economic pressure, and also related with the little number of patients in this experiment. The same phenomenon occurred in the BMS group. Pati [16] found OLP more occurred in adults, 50 to 55 years old female, male: female about 0.5:1. In this experiment OLP patients’ average age was 47.22, with the ratio of male and female was 0.61:1, The data was consistent with Pati’s, although low morbidity age than that, but in accordance with the clinical characteristics of multiple born in middle-aged and old women.

The purpose of this study was to investigate the psychological problems and oral health-related quality of life (OHRQoL) in patients with RAU, OLP, BMS. Measure instruments we chose were Oral Health Impact Profile (OHIP-14) to measure self-reported dysfunction, discomfort and disability attributed to oral conditions [6, 12], and Hospital Anxiety and Depression Scale (HADS) to test anxiety and depression in a clinical [13, 14].

In this study, it was found that nearly half of the patients in the experimental group had anxiety or depressive symptoms. This was how stress performed on them, and stress referred to different types of life experiences and personal reactions to them [17]. Everyone in life came under some kind of stress. In some people, the reactions to stress could lead to better adapt, but in other ways, it could lead to poor adaptation and the disease, causing anxiety or depression. The patients in the RAU, OLP and BMS group had different levels of anxiety and depressive symptoms. This suggested that psychological stress plays an important role in these diseases.

In the study, it was obvious that depression was closely at the RAU, and that not only anxiety but also depression had relationship with OLP and BMS, especially in BMS group. Similar conclusion could be got from the previous literature, like Zadik Y [18], Lidia Gavic1 [19] and Variously K [20]. There was a conclusion that a high correlation between anxiety, depression, and psychological stress with symptoms of RAS and OLP had been observed. The same conclusions from the BMS group were from Sousa FT [21]. Alternatively, it was observed anxiety and depression which were closely with BMS. Schiavone V [22] got the result that pain was affected by depression, and depression was affected by anxiety. BMS patients had statistically significant higher scores of anxiety and depression than the control group. There existed different points about the relation of BMS with psychological problems. The symptoms of anxiety, depression, fear were confirmed in this study. It can be seen that RAU, OLP, and BMS are related to the state of anxiety and depression, suggesting that patients may be accompanied by symptoms of anxiety or depression. The experimental results remind doctors to pay attention to the patient’s psychological problems in the clinical diagnosis and treatment process. Appropriate psychological counseling and psychotherapy can help improve the patient’s condition. But further research is needed to explain the interaction between psychological problems and disease.

From the comparison of scores from the RAU group, OLP group, BMS group and healthy control group, it could be concluded that the scores of physical pain had significant differences. In addition, RAU and OLP had poor quality of life, but there was no significant difference between BMS group and healthy controls. The difference in the physical pain domain confirmed the clinical character of pain in RAU, OLP and BMS, which suggested the importance of relieving the pain filling in treatment. Hupa A [11] found the pain relieved and the quality of life improved after the treatment. Zwiri AM [23] got the similar conclusion. In BMS group, the results might be due to the burning felling didn’t obstruct the sleeping and eating and didn’t influence the quality of life. However, Sousa TA [24] believed that BMS had a negative impact on an individual’s health-related quality of life. In summary, quality of life assessment might help to more information about the nature and severity of BMS, assessing the effectiveness of treatment options to improve outcomes through humanized clinical practice. In addition, we found significant differences in the social disability area of the OLP group. The results might be due to the OLP patient being overly concerned about the disease and not wanting to be associated with other patients under higher stress. The results of this study showed that the RAU group had the highest score, suggesting that the quality of life of RAU patients was the worst, which was related to the frequent recurrence of RAU, which significantly affected the clinical characteristics of eating. However, Lópezjornet P [6] obtained the highest score of OHIP-49 in BMS, and the scores of OLP group and RAU group were lower, which indicated that oral mucosal disease had an impact on patients’ health and quality of life. The reason for the inconsistency in the results of the two studies might be that the number of BMS patients included in this study was small, the study population was different, and the scales and statistical methods were different. However, the conclusions of the quality of life of patients with oral mucosal disease obtained by the two studies are consistent, suggesting that oral mucosal disease should reduce the quality of life of patients should be paid attention to. Therefore, it was necessary to advise patients to contact others and learn to relieve stress to improve their quality of life.

For most patients, oral mucosa diseases rarely endangered their life, but it had a serious effect on daily life. Anxiety and depression might be the result of oral mucosa diseases [25–27]. In 2003, Llewellyn [28] found that oral medicine outpatient clinic patients’ OHR-QoL scores were significantly lower than the general population OHR-QoL scores and overall OHR-QoL scores. Changes in anxiety, 55% were forecast by the general health rating and OHR-QoL field “psychological discomfort” and “psychological disability.” The variation in depression, 54% is predicted by the general health rating and the “functional limitations” and “social disability” in the OHR-QoL field. Positive correlations were found between the condition of anxiety and/or depression and quality of life of the patients with RAU, OLP, BMS in the present study. Yet there was no correlation between depression and quality of life in BMS. The reasons might be different study objects who came from different countries.

## Conclusion

Oral mucosal diseases such as RAU, OLP, BMS reduced the quality of life of patients. The anxiety/depression level of patients was higher than that of healthy people. The quality of life of patients was related to their psychological problems, suggesting that the decline in the quality of life of patients may affect the psychological state of patients. It was suggested that in the clinical diagnosis and treatment process, the psychological problems of patients with oral mucosal diseases need doctors to pay attention to.

## Abbreviations

BMS: Burning mouth syndrome
HADS: Hospital anxiety and depression scale
OHIP-14: Oral health impact profile
OHRQoL: Oral health-related quality of life
OLP: Oral lichen planus
RAU: Recurrent aphthous ulcers
